# Barriers to WHO prequalification of similar biotherapeutic insulin

**DOI:** 10.2471/BLT.24.291804

**Published:** 2024-09-25

**Authors:** Henry MJ Leng, Jicui Dong

**Affiliations:** a Innovation and Emerging Technologies Department, Access to Medicines and Health Products Division, World Health Organization, 20 Avenue Appia, 1211 Geneva, Switzerland.

## Abstract

**Objective:**

To identify the barriers preventing manufacturers of similar biotherapeutic human insulin from submitting their products to the World Health Organization (WHO) for prequalification.

**Methods:**

We used a self-administered questionnaire to collect data from companies producing similar biotherapeutic human insulin. We included questions about the insulin products manufactured, knowledge of WHO prequalification requirements, export of the products and compliance with good manufacturing practices. Companies had the possibility to provide additional relevant information. We sent the questionnaire to 20 manufacturers in total. We evaluated responses and organized the data into themes.

**Results:**

We had a response rate of 55% (11/20 companies). Five broad themes emerged: (i) manufacturers and products; (ii) expressions of interest awareness and participation; (iii) need for technical assistance and training; (iv) market and supply chain challenges; and (v) approval for good manufacturing practices. The most important reasons for manufacturers’ lack of response to WHO’s expression-of-interest invitation were absence of a mechanism to guarantee return on investment, and perceived complexity of prequalification requirements for insulin-similar biotherapeutic products.

**Conclusion:**

To encourage greater participation in the WHO prequalification programme, international procurement agencies associated with the programme should consider establishing a platform to enter into advance purchasing agreements with manufacturers. In addition, WHO’s Local Production and Assistance Unit should provide companies with ongoing technical assistance on the development of their human insulin products and improvement of their production facilities to comply with the WHO requirements for good manufacturing practices.

## Résumé

**Objectif:**

Identifier les obstacles qui empêchent les fabricants d'insuline biothérapeutique similaire à l'insuline humaine de soumettre leurs produits à l'Organisation mondiale de la Santé (OMS) en vue d'une préqualification.

**Méthodes:**

Nous avons utilisé un questionnaire auto-administré afin de récolter des informations auprès d'entreprises produisant de l'insuline biothérapeutique similaire à l'insuline humaine. Nos questions portaient notamment sur les types d'insuline fabriqués, les connaissances relatives aux conditions de préqualification de l'OMS, l'exportation des produits et le respect des bonnes pratiques de fabrication. Les entreprises avaient la possibilité de fournir tout renseignement complémentaire jugé pertinent. Nous avons envoyé le questionnaire à 20 fabricants au total, puis évalué les réponses obtenues et classé les données selon différents thèmes.

**Résultats:**

Le taux de participation a atteint les 55% (11/20 entreprises). Cinq grands thèmes en sont ressortis: (i) fabricants et produits; (ii) connaissance des manifestations d'intérêt et participation; (iii) besoins en matière d'assistance technique et de formation; (iv) difficultés au niveau des chaînes d'approvisionnement et du marché; et enfin, (v) approbation des bonnes pratiques de fabrication. Les principales raisons pour lesquelles les fabricants ne répondaient pas aux appels à manifestation d'intérêt de l'OMS étaient l'absence de mécanisme garantissant un retour sur investissement, ainsi que l'apparente complexité des conditions de préqualification pour les insulines biothérapeutiques similaires.

**Conclusion:**

Pour encourager la participation au programme de préqualification de l'OMS, les organismes acheteurs internationaux qui y sont associés devraient envisager de créer une plateforme permettant d'établir des conventions d'achat préalables avec les fabricants. En outre, l'Unité de Production Locale et d'Assistance (LPA) de l'OMS devrait fournir un support technique aux entreprises pour développer leurs insulines humaines et améliorer leurs installations de production afin de répondre aux exigences imposées par l'OMS en ce qui concerne les pratiques de fabrication.

## Resumen

**Objetivo:**

Identificar los obstáculos que impiden a los fabricantes de insulina humana bioterapéutica similar presentar sus productos a la Organización Mundial de la Salud (OMS) para su precalificación.

**Métodos:**

Se utilizó un cuestionario autoadministrado para recabar datos de empresas productoras de insulina humana bioterapéutica similar. Se incluyeron preguntas sobre los productos de insulina fabricados, el conocimiento de los requisitos de precalificación de la OMS, la exportación de los productos y el cumplimiento de las buenas prácticas de fabricación. Las empresas tenían la posibilidad de facilitar información adicional pertinente. Se envió el cuestionario a 20 fabricantes en total. Luego, se evaluaron las respuestas y se organizaron los datos por temas.

**Resultados:**

Se obtuvo una tasa de respuesta del 55% (11/20 empresas). Surgieron cinco temas generales: (i) fabricantes y productos, (ii) manifestaciones de interés concienciación y participación; (iii) necesidad de asistencia técnica y formación; (iv) desafíos del mercado y la cadena de suministro; y (v) aprobación de buenas prácticas de fabricación. Las razones más importantes de la falta de respuesta de los fabricantes a la invitación de la OMS a expresar su interés fueron la ausencia de un mecanismo que garantizara el retorno de la inversión y la percepción de la complejidad de los requisitos de precalificación para productos bioterapéuticos similares de insulina.

**Conclusión:**

Para fomentar una mayor participación en el programa de precalificación de la OMS, los organismos internacionales de adquisición asociados al programa deberían considerar la posibilidad de establecer una plataforma para celebrar acuerdos de compra anticipada con los fabricantes. Asimismo, la Unidad de Producción y Asistencia Local de la OMS debería proporcionar a las empresas asistencia técnica continua sobre el desarrollo de sus productos de insulina humana y la mejora de sus instalaciones de producción para que cumplan los requisitos de la OMS en materia de buenas prácticas de fabricación.

## ملخص

**الغرض:**

تحديد العوائق التي تمنع جهات تصنيع الأنسولين البشري العلاجي الحيوي المماثل من تقديم منتجاتها لمنظمة الصحة العالمية (WHO) بغرض التأهيل المسبق.

**الطريقة:**

لقد استخدمنا استبيانًا ذاتيًا لجمع البيانات من الشركات التي تنتج الأنسولين البشري العلاجي الحيوي المماثل. وقمنا بتضمين أسئلة حول منتجات الأنسولين التي تم تصنيعها، ومعرفة متطلبات التأهيل المسبق لمنظمة الصحة العالمية، وتصدير المنتجات، والامتثال لممارسات التصنيع الجيدة. كان لدى الشركات إمكانية تقديم معلومات إضافية ذات صلة. قمنا بإرسال الاستبيان إلى إجمالي 20 من جهات التصنيع، وقمنا بتقييم الاستجابات وتنظيم البيانات حسب الموضوعات.

**النتائج:**

حققنا معدل استجابة بنسبة %55 (11/20 شركة). ظهرت خمسة موضوعات رئيسية: (أ) جهات والمنتجات، و(ب) التعبير عن الاهتمام والوعي والمشاركة؛ و(ج) الحاجة إلى المساعدة الفنية والتدريب؛ و(د) تحديات السوق وسلسلة التوريد؛ و(هـ) الموافقة على ممارسات التصنيع الجيدة. كانت أهم الأسباب وراء عدم استجابة جهات التصنيع لدعوة منظمة الصحة العالمية للتعبير عن الاهتمام، هي غياب آلية ضمان العائد على الاستثمار، والتعقيد الملحوظ لمتطلبات التأهيل المسبق للمنتجات العلاجية الحيوية المشابهة للأنسولين.

**الاستنتاج:**

لتشجيع المزيد من المشاركة في برنامج التأهيل المسبق لمنظمة الصحة العالمية، يجب على هيئات المشتريات الدولية المرتبطة بالبرنامج أن تفكر في إنشاء منصة لإبرام اتفاقيات شراء مسبقة مع جهات التصنيع. بالإضافة إلى ذلك، يجب على وحدة الإنتاج والمساعدة المحلية لدى منظمة الصحة العالمية أن تقدم للشركات المساعدة الفنية المستمرة في تطوير منتجات الأنسولين البشري لديها وتحسين مرافق إنتاجها بهدف الامتثال لمتطلبات منظمة الصحة العالمية لممارسات التصنيع الجيدة.

## 摘要

**目的:**

确定人胰岛素生物仿制药生产商在向世界卫生组织 (WHO) 提交其产品以进行预认证方面存在的障碍。

**方法:**

我们采用自填问卷的方法从生产人胰岛素生物仿制药的公司收集相关数据。问卷纳入了有关所生产的胰岛素产品、对 WHO 预认证要求的了解、产品出口和遵守良好生产规范的问题。这些公司还可补充提供相关信息。我们总共向 20 家生产商发送了问卷。我们对回复情况进行了评估，并将数据按主题进行了分组。

**结果:**

问卷的回复率为 55%（20 家公司中有 11 家进行了回复）。共分为五大主题：(i) 生产商和产品；(ii) 了解和参与意向表达；(iii) 技术援助和培训需求；(iv) 市场和供应链挑战；以及 (v) 对良好生产规范的认可。生产商未回复 WHO 的意向表达邀请的最重要原因是缺乏保证投资回报率的机制，还有就是认为胰岛素生物仿制药的预认证要求太复杂。

**结论:**

为鼓励更多的生产商参与 WHO 的预认证项目，与该项目相关的国际采购商应考虑建立一个平台，用于与生产商签订预采购协议。此外，WHO 的本地化生产和援助部门应就人胰岛素产品的开发和生产设施的改进向公司持续提供技术援助，从而使其遵守 WHO 的良好生产规范相关要求。

## Резюме

**Цель:**

Выявить препятствия, которые мешают производителям аналогичных биотерапевтических препаратов человеческого инсулина представлять свою продукцию Всемирной организации здравоохранения (ВОЗ) для проведения преквалификации.

**Методы:**

Для сбора информации о компаниях, производящих аналогичный биотерапевтический препарат человеческого инсулина, использовались анкеты самостоятельного заполнения. Они включали вопросы о производимых инсулиновых препаратах, знании требований ВОЗ по преквалификации, экспорте продукции и соблюдении надлежащей производственной практики. Кроме того, компании имели возможность предоставить дополнительную информацию. В общей сложности анкеты были разосланы 20 производителям. Полученные ответы были обработаны и разделены на темы.

**Результаты:**

Доля ответивших составила 55% (11 из 20 компаний). Было выделено пять основных тем: (i) производители и продукция; (ii) выражение заинтересованности в осведомленности и участии; (iii) потребность в технической помощи и обучении; (iv) проблемы рынка и цепочки поставок; (v) утверждение надлежащей производственной практики. К наиболее важным причинам, по которым производители не откликнулись на предложение ВОЗ о выражении заинтересованности, относятся отсутствие механизма, гарантирующего возврат инвестиций, и предполагаемая сложность преквалификационных требований к аналогичным биотерапевтическим препаратам инсулина.

**Вывод:**

Для стимулирования более широкого участия в программе преквалификации ВОЗ международным закупочным организациям, связанным с этой программой, следует рассмотреть возможность создания платформы для заключения предварительных соглашений о закупках с производителями. Кроме того, местное подразделение ВОЗ по производству и оказанию помощи должно предоставлять компаниям постоянную техническую помощь в разработке ими препаратов человеческого инсулина и совершенствовании производственных мощностей в соответствии с требованиями ВОЗ к надлежащей производственной практике.

## Introduction

Despite advances in diabetes management, many people with diabetes still lack access to insulin.^1^^–^^4^ Contributing factors include market dominance by three multinational manufacturers; limited competition from insulin biosimilars (also referred to as similar biotherapeutic products, defined as biological products that are highly similar in quality, safety and efficacy to an already licensed reference product); logistical problems affecting supply security; differences in regulatory frameworks for similar biotherapeutic products; market shifts favouring more expensive insulin analogues (structurally modified insulins such as insulin glargine and insulin aspart); and insufficient local production.^5^^–^^11^

Insulin underuse is widespread in countries that have no universal health-care coverage.^12^ A recent study found that high mortality rates from diabetes in low- and middle-income countries are often due to out-of-pocket insulin costs.^12^^,^^13^ Lack of insulin access is the leading cause of death in children with type 1 diabetes in sub-Saharan Africa, and some patients may live just 1 year after diagnosis due to intermittent insulin availability.^14^^–^^17^ If access to basal human insulin in these countries is unaffordable, then replacing it with higher-priced insulin analogues could exacerbate the problem, as seen in higher-income countries. Because of the large price difference and the perceived benefit not justifying the cost, opposition was raised against including insulin analogues in the 2019 *World Health Organization Model list for essential medicines*.^5^

Affordability is crucial for access to insulin.^18^ The evolution of insulin – from animal to human forms and present-day insulin analogues – has led to increased prices, making it difficult for many people with diabetes even in high-income countries to afford the medication.^19^ In the United States of America, some patients are forced to ration their insulin use, leading to higher morbidity and even mortality.^12^^,^^20^^–^^22^

On 13 November 2019, the World Health Organization (WHO) invited manufacturers of human insulin products to submit an expression of interest for evaluation of these products. By February 2020, WHO had launched a pilot procedure to prequalify these products after evaluation for quality, safety and efficacy to increase availability in low- and middle-income countries.^23^ However, with no products submitted and hence prequalified after 2 years, WHO issued a second call for expression of interest on 17 May 2022. This call included evaluations of long-acting insulin analogues, insulin degludec, insulin detemir and insulin glargine as therapeutic alternatives.^24^

On 22 September 2022, WHO prequalified four human insulin biotherapeutic products, which were all innovator products submitted by Novo Nordisk (Bagsværd, Denmark). These products included solutions (100 IU/mL) or suspensions (100 IU/mL), both for injection in vials and cartridges. To date, no human insulin-similar biotherapeutic products have been prequalified, nor are any in the prequalification pipeline. This situation is unusual, as WHO prequalification offers advantages to manufacturers, such as opening up access to international markets, including procurement by United Nations agencies; faster registration through a shorter process as many national regulatory authorities recognize the WHO prequalification programme; and enhanced credibility for the manufacturer as WHO prequalification is a mark of quality, safety and efficacy. Furthermore, companies can make a global health impact as they will be providing essential medicines that meet international standards.^25^ The reluctance of manufacturers therefore suggests unique factors that may have hindered participation in the WHO pilot programme.^23^

We aimed therefore to identify the barriers that prevent manufacturers of human insulin-similar biotherapeutic products from submitting applications to the WHO prequalification programme. We also wanted to explore ways to encourage their response to WHO’s invitation of an expression of interest for product evaluation.

## Methods

### Questionnaire design

We used a self-administered questionnaire in two stages to identify barriers preventing manufacturers of human insulin-similar biotherapeutic products from participating in the WHO prequalification programme. In the initial stage, we compiled items based on literature reviews and team discussions.

The first draft of the questionnaire included two sections. Section 1 had 20 closed-ended questions on the following areas: (i) the types of insulin products manufactured and whether they included the products listed in WHO’s invitation in November 2019; (ii) knowledge of WHO prequalification requirements for similar biotherapeutic products; (iii) whether the products were supplied only locally or also exported; and (iv) whether the company had been inspected for compliance with good manufacturing practices by the regulatory authorities of countries to which the products were exported. Section 2 had open-ended questions that allowed companies to provide additional relevant information or describe issues not covered in the survey.

The questionnaire began with an explanation of its purpose and a metadata section for companies to enter basic information such as company name, address, website, year established and number of employees. The survey concluded with an invitation for companies to contact the investigators with any questions.

The first version of the questionnaire was pretested among 10 insulin manufacturers, but only five responded and none answered the open-ended questions. After clarifying misunderstandings, we rephrased some closed-ended questions to allow for opinions and justifications. We kept the section with open-ended questions as it was. We then sent the second version to 15 companies who had not participated in the pilot – 10 which had not been sent the first pilot version, and five which had been sent the first version but had not responded.

To maximize the response rate, we sent the questionnaire by email and sent weekly reminders. We also contacted national regulatory authorities or professional pharmaceutical manufacturing associations and, in one case, an embassy for assistance to encourage response. 

### Insulin manufacturers

To identify potential respondents, we used a list from Health Action International to find companies producing biosimilar insulin drug substances (active biological ingredient or insulin molecules which occur as crystals) or final products (finished pharmaceutical product in a vial or prefilled syringe).^26^ The final list included 20 companies. We excluded innovator insulin producers (for example, Novo Nordisk, Eli Lilly and Sanofi) and their subsidiaries, as well as subsidiaries of companies producing similar biotherapeutic products in countries other than their country of origin. 

### Data analysis

We analysed the qualitative data using framework analysis, a thematic analysis method that combines deductive coding with predefined themes and inductive coding to generate new themes.^27^^–^^29^ We used Quirkos version 2.5.2 (Quirkos Software, Edinburgh, Scotland) for data management and coding. We collected descriptive data, including date of establishment of the company, insulin manufacturing experience and number of employees, using the Quirkos tool.

## Results

Our response rate was 55% (11/20 companies). We coded responses from both versions of the questionnaire. Before coding, we used expert judgement to assess rephrased questions from the second version for differences in meaning from the first version but found no differences. We grouped the codes, based on individual questions, into five broad themes: (i) manufacturers and products, that is geographic location of companies, the maturity level of the regulatory authorities^30^ of the countries in which the companies are located, their insulin product range and their manufacturing experience; (ii) expressions of interest awareness and participation, that is knowledge of WHO’s calls and response to them; (iii) technical assistance and training, that is the need for capacity-building by WHO to help companies understand the guidelines on and requirements of WHO’s prequalification of insulin-similar biotherapeutic products; (iv) market and supply chain challenges, that is the regulatory and logistical challenges companies encounter when exporting their insulin products to other, mainly developing, countries; and (v) good manufacturing practice approvals, that is compliance with requirements for good manufacturing practices among companies that export their products to foreign countries.

These themes highlight the main obstacles and concerns identified by the respondents as barriers to their participation in the WHO prequalification programme for human insulin.

### Manufacturers and products

The companies were all manufacturers of similar biotherapeutic products from various regions. Companies were based in Egypt, India, Morocco, Poland and the Russian Federation (all one each) and Bangladesh and China (both three each). After an assessment by WHO, we considered the national regulatory authorities of several countries, including China, Egypt and India, to have maturity level three (on a scale of one to four) for vaccines. This level indicates that these countries have established stable, well-functioning and integrated regulatory systems.^31^

The experience of these companies in insulin manufacturing varied, ranging from 3 to 40 years, with nine companies having more than a decade of experience.

Seven companies manufacture both insulin crystals (the drug substance) and the drug product. The other companies source the drug substance from third-party suppliers outside the country in which the drug product is manufactured, which typically limits the markets the company can supply. One such company initially restricted its sales to its domestic market, but was later allowed to export on a case-by-case basis by the drug substance supplier, which also marketed its own drug product in several countries. This company, with strong research and development capabilities in biosimilars, attempted to produce the insulin drug substance itself but found it financially unfeasible due to high costs. Additionally, the company lacked market insight on forecasted volumes required by procurement agencies, which would help in decision-making.

**Fig. 1:**
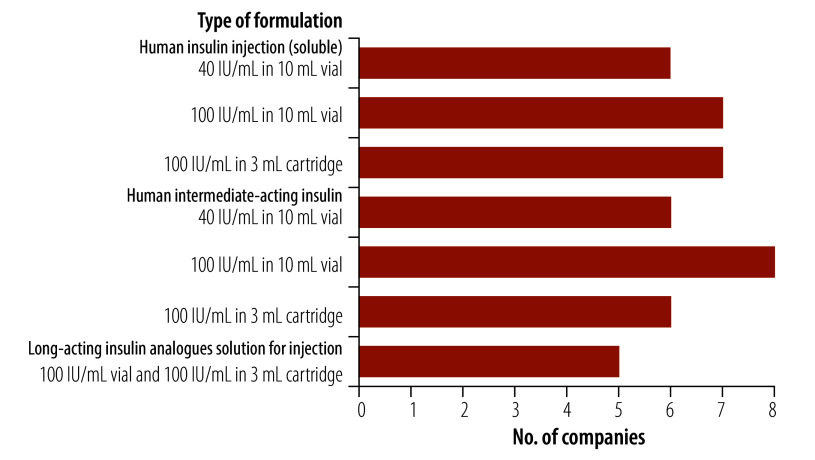
Number of companies manufacturing human insulin products listed in WHO’s first expression-of-interest invitation, 2019

Nine companies manufacture at least one of the four insulin presentations listed in the first invitation for expression of interest (Fig. 1), with four companies producing all four presentations. One company, producing only the two 100 IU/mL vial presentations, can also manufacture the equivalent 40 IU/mL strengths. Another company stopped producing the human insulin presentations listed in the call for expression of interest after losing a government tender. A company manufacturing only analogue insulin stated that it would not produce human insulin as its government had finalized its insulin purchases. The reasons given for not manufacturing human insulin included the unpopularity of vials, patient preference for long-acting analogue insulins and the high market value of these analogue insulins.

WHO: World Health Organization.

Note: Long-acting insulin analogues were added to the second expression-of-interest invitation.

### Expression of interest awareness

**Fig. 2:**
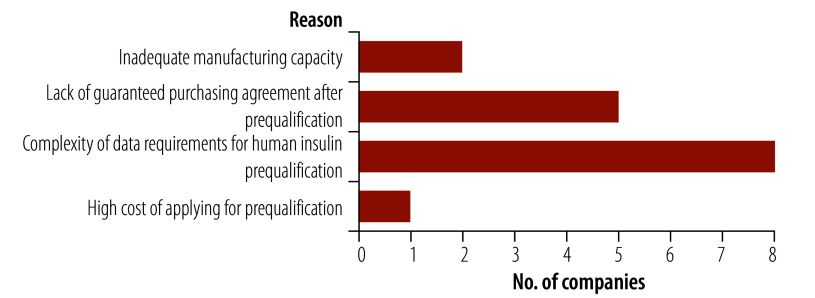
Reasons companies did not participate in WHO’s expression-of-interest invitation, 2019

Of the 11 companies, seven were aware of the WHO invitation for expression of interest, and one of the companies participated in the workshops held by the prequalification team on the first expression-of-interest invitation. However, none of the companies applied for prequalification of their insulin products for several reasons including: lack of manufacturing capacity; perceived high cost of prequalification; complex data requirements for prequalification of insulin and other similar biotherapeutic products; and absence of purchasing agreements from international procurement agencies (Fig. 2). Additionally, one company said that WHO did not provide detailed information on procurement volume and price, making it difficult to evaluate the business case.

WHO: World Health Organization.

Box 1 lists recommendations made by responding companies that might encourage them to participate in the insulin prequalification programme. One company thought that incentives would help them proceed with the prequalification proposal and “bind the company to emphasize the project.” Seven companies indicated that they would take part in the second expression-of-interest invitation, which included requests for both human insulin and long-acting insulin analogue products. Some companies said they would submit only human insulin or analogue insulin formulations, while other companies indicated they would submit both. A company that could not participate in the first expression of interest due to insufficient manufacturing capacity is developing a state-of-the-art facility and is “…very much interested to apply for WHO prequalification of human insulin and insulin analogues.”

Box 1 **Recommendations of respondents to encourage their participation in WHO’s programme for the prequalification of human insulin products**

- Practical training on, and technical assistance with, data requirements for the prequalification of human insulin and other biotherapeutics.
- Simulated inspections on good manufacturing practices to identify gaps in compliance before WHO prequalification inspections.
- Financial assistance for comparative clinical trials to demonstrate biosimilarity with the innovator human insulin product.
- Guaranteed product volumes from procurement agencies for 3 to 5 years to better plan manufacturing capacity.
- Allowance to adjust prices for inflation and geopolitical events to maintain profitability.

WHO: World Health Organization.

### Technical assistance

Ten companies requested training and technical assistance to understand WHO’s requirements for the prequalification of insulin-similar biotherapeutic products, compile the common technical document or product dossier, and meet WHO’s current requirements for good manufacturing practices. The companies also sought support in the following areas: (i) reviewing existing product formulations and assessing their suitability; (ii) extrapolating clinical data; (iii) validating transport systems and management of cool chains, especially in hot climates; (iv) undertaking stability studies of similar biotherapeutic products; (v) training in pharmacovigilance; (vi) developing product leaflets, patient information leaflets and material safety data sheets; (vii) guidance on nonclinical and clinical data requirements; and (viii) guidance on data requirements for the chemistry, manufacturing and control of similar biotherapeutic products.

Although all the insulin products of the companies are authorized for marketing in their own countries, more than half were uncertain whether their product dossiers would comply with WHO’s prequalification requirements for similar biotherapeutic products. 

### Market challenges

Seven companies export their products to foreign countries. However, most importing countries do not have stringent regulatory authorities, such as in North America, the European Union, Australia, Japan, and United Kingdom of Great Britain and Northern Ireland.^32^ The importing countries are spread across Africa, Asia, the Middle East and South America. In some of these countries, including those where some respondent companies are located, human insulin products are licensed as either biosimilars (as biological medicines) or generics (as pharmaceutical medicines). Difficulties associated with exporting included freight logistics, cold chain delivery, small minimum order quantities, long market approval processes, different regulations in international markets, and low selling prices in African countries. One responder said that many countries, even those outside the European Union or United States, require companies to be certified by the European Medicines Agency (EMA) or United States Food and Drug Administration (FDA) for their manufacturing site. This certification cannot be obtained without applying for authorization in those more established markets. Without such certification, licensing in these countries is a costly and time-consuming process.

### Good manufacturing practices

Nine companies are licensed by foreign national regulatory authorities for compliance with good manufacturing practices, but not all importing countries have conducted site inspections for good manufacturing practices. Only two companies have been inspected by the United States FDA or by both FDA and EMA. Additionally, one company has an insulin analogue product authorized in both Europe and the United States. Two companies have WHO-prequalified products, indicating that their manufacturing premises comply with WHO’s requirements for good manufacturing practices.

## Discussion

We aimed to identify the reasons for the non-response of companies producing insulin products to WHO’s invitation for prequalification, and suggest ways to encourage manufacturers to seek prequalification. Although most companies were aware of WHO’s first invitation for expression of interest, none participated. The reasons included the absence of guaranteed purchasing agreements for prequalified products, and the perceived high cost and complexity of prequalification procedures. However, prequalification of biotherapeutic products, including insulin, does not incur fees as it is a pilot project,^24^ and manufacturers need to be informed that prequalification of biotherapeutics currently carries no costs. Furthermore, as most of the companies were uncertain whether their product dossiers complied with WHO’s prequalification requirements for similar biotherapeutic products, there is a clear need to educate manufacturers on the data requirements for prequalifying human insulin products.

The companies gave several recommendations (Box 1) that might encourage them to participate in prequalification. Two recommendations – providing technical assistance with the clinical and quality data requirements, and conducting simulated onsite inspections for good manufacturing practices to identify compliance gaps before official WHO inspections – can be addressed by the WHO Local Production and Assistance Unit in the WHO Department of Innovation and Emerging Technologies. This unit supports companies, especially in low- and middle-income countries, with technical assistance, training and dossier preparation to accelerate WHO prequalification.^33^ The WHO prequalification team in the Regulation and Prequalification Department also has annual workshops on the prequalification of biotherapeutic products to address quality, clinical and biosimilarity data requirements.^34^

Other proposed incentives included guaranteed purchasing quantities, authorization to adjust prices for inflation and geopolitical events, and financial assistance for clinical trials. Guaranteed purchasing quantities or agreements and price adjustments can be negotiated with purchasing agents. Financial assistance relates to the clinical studies required for prequalification of similar biotherapeutic products of insulin.

The prequalification requirements for similar biotherapeutic products are described in the WHO guidelines on evaluating similar biotherapeutic products, which were amended in 2020 with an expanded scope, updated pharmacovigilance sections and revised evaluation criteria.^35^ For human insulin-similar biotherapeutic products, data requirements are fewer than similar biotherapeutic products of rituximab and trastuzumab, as only a comparative pharmacokinetic and pharmacodynamic assessment against the reference biotherapeutic product in healthy people is required.^23^^,^^24^ In contrast, a comparative phase III trial is mandatory for the other similar biotherapeutic products, including for insulin analogues. This trial is in addition to comparative physicochemical and in vitro biological data that must be submitted for all similar biotherapeutic products to support a claim of biosimilarity.^35^

The costs of developing a biosimilar product range from 100 million United States dollars (US$) to US$ 300 million, with clinical studies accounting for 65% of this cost.^36^ The cost will be less for human insulin-similar biotherapeutic products as phase III trials are not required. Nevertheless, the remaining cost is still high compared with developing a generic medicine, which costs between US$ 5 million and US$ 15 million.^37^ Our survey results suggest that insulin’s limited profitability makes the US$ 100 million investment not financially viable for many manufacturers in low- and middle-income countries. Consequently, some manufacturers have shifted to producing insulin analogues.

Most companies had marketing authorization for their products in low- and middle-income countries with less-established national regulatory authorities. Respondents noted that licensing their products as generics required significantly fewer data than for a similar biotherapeutic product. For instance, a generic medicine formulated as a solution for injection, such as soluble human insulin injection, could qualify for a biowaiver, exempting the company from submitting a human bioequivalence or comparative pharmacokinetic study. Comparative physicochemical and in vitro biological data were also unnecessary, as such data are not required for conventional generic medicines.

The WHO prequalification team has established an alternative prequalification pathway for human insulin products from manufacturers in countries without stringent regulatory authorities. These so-called standalone applications are accepted if they include a comparative pharmacokinetic and pharmacodynamic study against a reference biotherapeutic product from a market with a stringent regulatory authority and a 6-month comparative safety trial. Companies may have conducted a pharmacokinetic and pharmacodynamic study for complex intermediate-acting insulins, but it is unlikely they would have performed a 6-month safety study before seeking authorization in developing countries.

The United States FDA has acknowledged that the slow pace of biosimilar submissions is due to the time and cost of producing the required data packages. To overcome this problem, FDA has launched a regulatory science programme under the Biosimilar User Fee Act (BsUFA III) for fiscal years 2023 to 2027.^38^ This programme aims to advance the development of interchangeable products (that is, from original biological product to a biosimilar medicine) and improve the efficiency of biosimilar product development. FDA is exploring the use of real-world evidence, such as health data from electronic health records and registries, to support interchangeability decisions, instead of requiring switching clinical trials.^38^

Could a similar approach be applied to the assessment of standalone insulin products using real-world data? If such data are unavailable or insufficient, could pharmacovigilance and periodic safety update reports from post-marketing surveillance in countries where these insulin products are marketed serve a similar purpose? This approach could potentially reduce the need for costly comparative safety trials.

The empirical results reported in our study should be considered against the following limitations. First, we only looked at manufacturers of similar biotherapeutic human insulin; hence, the findings cannot be generalized to manufacturers of innovator human insulin products. Second, we used a self-administered questionnaire, which can gather valuable information, although not to the same depth as other methods such as interviews or focus groups. Additionally, response bias is a risk, given that a small number of respondents opted not to answer specific questions. Third, our response rate of 55% is low; however, for online surveys, a response rate of 44% and higher is considered acceptable.^39^

In conclusion, most of the manufacturers surveyed view conventional human insulin products as low value compared with insulin analogues. As a result, they are reluctant to invest in further development to comply with WHO prequalification requirements without guaranteed returns. Many low- and middle-income countries have human insulin manufacturers with more than a decade of experience, which meets local regulatory standards. To facilitate prequalification of standalone human insulin products without a 6-month comparative safety study, alternative regulatory approaches could be considered, including production history and compliance with good manufacturing practices. Additionally, the training and specialized technical assistance offered to companies in low- and middle-income countries by the WHO Local Production and Assistance Unit should be more widely promoted to expedite prequalification.

Finally, our study suggests that an opportunity exists for WHO to develop a framework for the prequalification of similar biotherapeutic products for developing countries. This framework could incorporate real-world data as substitute evidence to fill any gaps in current prequalification requirements. Notably, some regulatory authorities have begun exploring the use of real-world data for regulatory decision-making.^40^^,^^41^
